# EXPRESSION OF E-CADHERIN AND WNT PATHWAY PROTEINS BETACATENIN, APC, TCF-4 AND SURVIVIN IN GASTRIC ADENOCARCINOMA: CLINICAL AND PATHOLOGICAL IMPLICATION

**DOI:** 10.1590/0102-6720201600040004

**Published:** 2016

**Authors:** Rodrigo Rego LINS, Celina Tizuko Fujiyama OSHIMA, Levindo Alves de OLIVEIRA, Marcelo Souza SILVA, Ana Maria Amaral Antonio MADER, Jaques WAISBERG

**Affiliations:** 1 Postgraduate Program in Interdisciplinary Surgical Science, Federal University of São Paulo - UNIFESP, São Paulo, SP; Brazil; 2 Department of Pathology, UNIFESP, São Paulo, SP, Brazil; 3 Discipline of Pathology, ABC Medical School, Santo André, SP, Brazil.

**Keywords:** Wnt signaling pathway, Beta catenin, Stomach neoplasms, Cadherins, Immunohistochemistry

## Abstract

**Background:**

Gastric cancer is the fifth most frequent cancer and the third most common cause of cancer-related deaths worldwide.It has been reported that Wnt/ betacatenin pathway is activated in 30-50% of these tumors. However,the deregulation of this pathway has not been fully elucidated.

**Aim:**

To determine the expression of E-cadherin, betacatenin, APC, TCF-4 and survivin proteins in gastric adenocarcinoma tissues and correlate with clinical and pathological parameters.

**Method:**

Seventy-one patients with gastric adenocarcinoma undergoing gastrectomy were enrolled. The expression of E-cadherin, betacatenin, APC, TCF-4 and survivin proteins was detected by immunohistochemistryand related to the clinical and pathological parameters.

**Results:**

The expression rates of E-cadherin in the membrane was 3%; betacatenin in the cytoplasm and nucleus were 23,4% and 3,1% respectively; APC in the cytoplasm was 94,6%; TCF-4 in the nucleus was 19,4%; and survivin in the nucleus 93,9%. The expression rate of E-cadherin was correlated with older patients (p=0,007), while betacatenin with tumors <5 cm (p=0,041) and APC with proximal tumors (p=0,047). Moreover, the expression of TCF-4 was significantly higher in the diffuse type (p=0,017) and T4 tumors (p=0,002).

**Conclusion:**

The Wnt/betacatenin is not involved in gastric carcinogenesis. However, the high frequency of survivin allows to suggest that other signaling pathways must be involved in the transformation of gastric tissue.

## RESUMO

**Racional:**

O câncer gástrico encontra-se entre as principais neoplasias malignas do mundo sendo o quinto mais incidente e o terceiro em relação ao índice de mortalidade. Acredita-se que a via Wnt/betacatenina esteja ativada em 30-50% desses tumores, porém a desregulação dela ainda não está completamente esclarecida.

**Objetivo:**

Avaliar a imunoexpressão das proteínas E-caderina, betacatenina, APC, TCF-4 e survivina em tecidos de adenocarcinoma gástrico e correlacioná-las com as variáveis clínicas dos doentes e anatomopatológicas do tumor.

**Método:**

Foram coletados os dados clínicos e anatomopatológicos dos prontuários de 71 doentes com adenocarcinoma gástrico submetidos à gastrectomia. O material obtido na operação foi submetido à análise imunoistoquímica e a frequência da expressão de cada proteína pôde ser analisada de acordo com a sua localização na célula e relacionada com as variáveis clinicopatológicas.

**Resultados:**

A graduação percentualda expressão e da localização das proteínas foi a seguinte: E-caderina em 3% na membrana; betacatenina em 23,4% no citoplasma e 3,1% no núcleo; APC em 94,6% no citoplasma; TCF-4 em19,4% no núcleo; e survivina em 93,9% no núcleo. Houve relação entre expressão da proteína E-caderina com a idade mais avançada (p=0,007); betacatenina com tumores <5 cm de diâmetro (p=0,041);APC com tumores proximais (p=0,047); e TCF-4 com tipo difuso da classificação de Lauren (p=0,017) e com o grau de penetração tumoral (p=0,002).

**Conclusão:**

A via Wnt/betacatenina não está envolvida na carcinogênese gástrica. Porém, a frequência elevada de survivina permite sugerir que outras vias sinalizadoras devam estar envolvidas na transformação do tecido gástrico.

## INTRODUCTION

Gastric cancer (GC) is the fifth most frequent cancer and the third most common cause of cancer-related deaths worldwide^11^. More than 70% of cases occurred in developing countries and Brazilian estimates for the year 2016 by the National Cancer Institute are 20,520 new cases^14^.

The complete resection is the only way to cure GC but approximately 75% of these patients are diagnosed at an advanced stage and cannot be cured merely by surgery, so chemotherapy combined with surgery is often required^5^. Despite recent advances in diagnostic methods, surgical techniques, chemotherapy regimens and targeted therapy, more than half recur^16^. In addition, the prognosis of patients with advanced GC remains relatively poor with a median overall survival of 12 months in Western countries^28^.

An understanding of the pathogenic mechanisms and the different pathways may be critical for improvements in the diagnosis, treatment or prediction of prognosis of GC. The progression into an invasive cancer is a complex multifactorial process with genetic and epigenetic changes affecting several signaling pathway such as Wnt/ betacatenin^15^. 

Wnt/betacatenin pathway is a conserved molecular system that plays a major role in embryogenesis and tissue homeostasis, as well as tumorigenesis^8^. The activity of this pathway is dependent on the amount of betacatenin in the cytoplasm. Normally, the level of cytoplasmic β-catenin is maintained low through ubiquitin-proteasome-mediated degradation, regulated by a multiprotein "destruction" complex containing the core protein axin, adenomatous polyposis coli (APC), glycogen synthase kinase-3beta (GSK-3beta) and casein kinase 1 (CK1)^17^. In addition, betacatenin can also be detected in the membrane, integrated with a transmembrane glycoprotein E-cadherin forming a complex that play a key role in the maintenance of cell/cell adhesion in epithelial tissues^13^. It well known that loss of E-cadherin expression enhances cell migration and promotes metastasis^9^. 

The binding of Wnt proteins to a membrane receptor complex comprised of Frizzled/low-density lipoprotein receptor-related protein (Fzd/LRP) initiates a signaling cascade, activating and recruiting disheveled (Dsh) and AXIN to the membrane, thereby disrupting the destruction complex. As a result, free no phosphorylated betacatenin accumulates in the cytoplasm and translocates into the nucleus. In the nucleus, β-catenin displaces Groucho, binds T cell factor/lymphoid enhancer factor (TCF/LEF) and initiates transcription of its target genes as survivin^25^. 

It has been reported that aberrant Wnt/ betacatenin signaling is widely implicated in diverse human malignancies including GC ^2^ ^,^ ^7^ ^,^ ^22^. The nuclear accumulation of betacatenin, a hallmark of Wnt signaling activation, is found in more than 50% of gastric cancers^6^. However, the mechanisms that promote deregulation of the Wnt pathway in GC are still not completely understood^10^.

To study the influence of Wnt in CG, was proposed to evaluate the expression of E-cadherin, betactenin, APC, TCF-4 and survivin proteins in gastric carcinoma tissues and correlate with clinico-pathological parameters.

## METHODS

The Ethic Review Committee at Universidade Federal de São Paulo (UNIFESP) approved the study protocol (Registration n^o^ 834.176).

### Patients 

A total of 74 specimens of primary gastric carcinomas (GC) were collected from patients who underwent radical surgical resection at the Department of General Surgery of Faculdade de Medicina ABC (SP, Brazil) from January 2007 to December 2010. The patients' medical records were reviewed to determine their age, gender, anatomical site, tumor size, histological grade and the presence or absence of lymphatic, vascular or neural invasion. The inclusion criteria were patients aged over 18 years, of both genders, whom had undergone curative or palliative gastrectomy without neoadjuvant radio or chemotherapy, with histological examination confirming gastric carcinoma. Three patients were excluded because their paraffin blocks were unsuitable for histopathology and immunohistochemistry.

### TMA construction

TMA blocks also called receptor block were constructed at the laboratory of Pathology Department of Faculdade de Medicina da Universidade de São Paulo. For this, the paraffin blocks containing the tissue of gastric cancer from the Department of Pathology of Faculdade de Medicina do ABC were used.

Representative areas selected by a pathologist of the 71 gastric carcinomas were selected from hematoxylin-eosin stained sections. The selected area was marked in the respective paraffin block. A cylindrical core was created in the receptor block using using Beecher(tm) equipment (Beecher Instruments, Silver Spring, MD, USA). A 1 mm cylinder of tissue was extracted from the selected area of the donating block and was transferred to the core in the receptor block. New core positions were created in the receptor block, separated by fractions of 1 mm such that a collection of tissue samples was created following the matrix arrangement.

### Immunohistochemistry

Immunohistochemistry methods were performed at Experimental Molecular Pathology Laboratory I Department of Pathology for evaluating the expression of E-cadherin, betacatenin, APC, TCF-4 and survivin.

Slides of 3 µm were obtained from TMA block and mounted pretreated with 3-minopropyl-triethoxysilane (Sigma, St. Louis, MO, USA). Sections were deparaffinized in three changes of xylene and hydrated in a graded series of ethanol finishing in distillated water. For antigen retrieval slides were placed in 0,01M citrate-buffer pH 6,0 and heated in a steamer for 30 min. Endogenous peroxidase was blocked by using 10% hydrogen peroxide for 20 min. The slides were then washed in distillated water and phosphate buffer saline (PBS) and incubated overnight with the following monoclonal igG antibodies: betacatenin (E5 sc-7963, dilution 1:200, Santa Cruz, California, USA); E-cadherin (H-108 sc-7870, dilution 1:100, Santa Cruz, California, USA); APC (C-20 sc-896, dilution 1:100, Santa Cruz Biotechnology, Santa Cruz, CA, USA); TCF-4 (H-125 sc-13027, dilution 1:100, Santa Cruz Biotechnology, Santa Cruz, CA, USA); survivin (FL-142 sc-10811, dilution 1:100, Santa Cruz Biotechnology, Santa Cruz, CA, USA). Subsequently, slides were washed with PBS and incubated with biotinylated secondary antibody for 30 min, washed with PBS, and incubated with streptavidin-biotin-peroxidase (LSAB kit, Dako, USA) for 30 min each.

Finally, the reaction was revealed using 3,3'-diaminobenzidine tetrahydrocloride (Kit DAB-Sigma, Sigma-Aldrich, St. Louis, MO, USA) and counterstained with Harris's hematoxylin and coverslipped with Entellan (Merck KGaA, Darmstadt, GER). Negative and positive controls were made to run simultaneously.

The positive pattern for E-cadherin (membrane and cytoplasm), betacatenin (membrane, cytoplasm, nucleus), APC (cytoplasm and nucleus), TCF-4 (cytoplasm and nucleus), survivin (cytoplasm and nucleus) were noted. As a positive control of the reactions, a histological cut of colon adenocarcinoma was used, which proved to be positive for the studied proteins.

### Evaluation

Two trained researchers evaluated the expression of each protein independently and they were blinded to the clinico-pathological parameters. They analyzed the percentage of positive cells and staining intensity. The percentage score of positive cells was classified semi-quantitatively as follows: 0, ≤10% of positive cells; 1, between 11-25% of positive cells; 2, between 26-50% of positive cells; and 3, >50% of positive cells. The intensity score was graded as follows: 0, no brown in the cells; 1, light brown in the cells; 2, brown in the cells; and 3, strong brown in the cells. The overall score (OS) was calculated by the multiplication of percentage score and the intensity score. A patient with OS higher or equal than 4 was defined as positive for the expression of the protein and those with scores less than 4 negative.

### Statistical analysis 

The relationships between the immunohistochemical status of E-cadherin, betacatenin, APC, TCF-4 and survivin with various clinico-pathological findings were evaluated. Continuous data were compared by Mann-Whitney U test. Categorical analysis of variables was performed by Fisher's exact test. A p<0.05 was considered statiscally significant.

## RESULTS

Of the 71 patients with gastric adenocarcinoma analyzed, 64.8% were men and 60.6% were older than 60 years.

The morphologic characteristics of gastric carcinoma are shown in Table 1. It was found that 63.4% of the tumors were located in the proximal region and that 54.3% had a diameter greater than 5 cm. Regarding the degree of cell differentiation, 55.9% were well or moderately differentiated, 69% were intestinal type and the presence of venous, lymphatic and perineural was found in 33.3%, 56.5% and 62.3 % of cases, respectively.

**TABLE 1:**
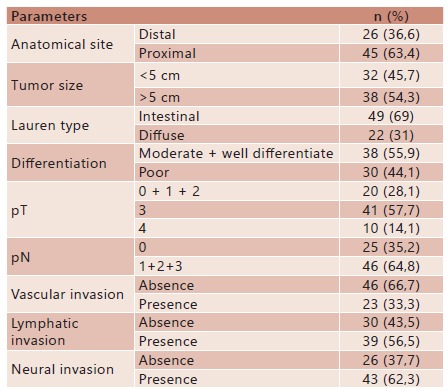
Morphologic characteristics of gastric carcinoma

n=number of cases

The frequency of immunostaining of studied proteins was: E-cadherin 3% in the membrane; betacatenina 29.7% in the membrane, 23.4% in the cytoplasm and 3.1% in the nucleus, APC 94.6% in cytoplasm, TCF-4 19.4% in nucleus and survivin 93.9% in nucleus (Table 2)

**TABLE 2:**
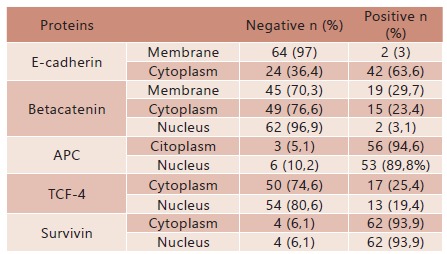
Immunohistochemical expression of E-cadherin, betacatenin, APC, TCF-4 and survivin in gastric cancer

n= number of cases

The relationship between the clinico-pathologic parameters and immunohistochemical expression of the proteins is exposed in Table 3. Regarding the expression of betacatenin in the membrane, more women were noted in the group of tumors with betacatenin positive than negative (p=0.024). Considering only the group of tumors with betacatenin in the membrane positive, there was 3.78 times more chance to involve women than men (p=0.018 [1.23-11.65]). For betacatenin expression in the cytoplasm, there was a greater number of tumors ≤5 cm in the group with positive expression of betacatenin than in the group with negative expression (p=0.041). Analyzing the group of tumors with positive expression of betacatenin in the cytoplasm, there was 3.65 times more chance to be ≤5 cm (p=0.034 [1.07 to 12.42]). At the nucleus, there was no significant association between betacatenin and clinico-pathological parameters. About APC in the cytoplasm, it was noted that all proximal tumors showed positive expression (p=0.047). In relation to TCF-4 in the nucleus, there was an association between positive expression and pT (p=0.002). Tumors with positive expression showed 12.00 times more chance to be pT4 (p=0.003 [1.79 to 80.61]) and 10.50 times more chance to be in the group pT0+ 1 + 2 (p=0.002 [1.91 to 57.59]) than to be pT3. Moreover, was noted the association with the diffuse type tumors (p=0.017) and the tumors with positive expression of TCF-4 in the nucleus, showed 5.05 times more likely to be diffuse type of Lauren than the intestinal type (p=0.009 [1.40 to 10.14]). Finally, it can be considered that there was a trend of the association between the positive expression of survivin and intestinal type of Lauren tumors (p=0.091), because tumors with positive expression of survivin in the nucleus and/or cytoplasm showed 7.33 times more likely to be intestinal type of Lauren (p=0.050 [0.71 to 75.27]).

**TABLE 3:**
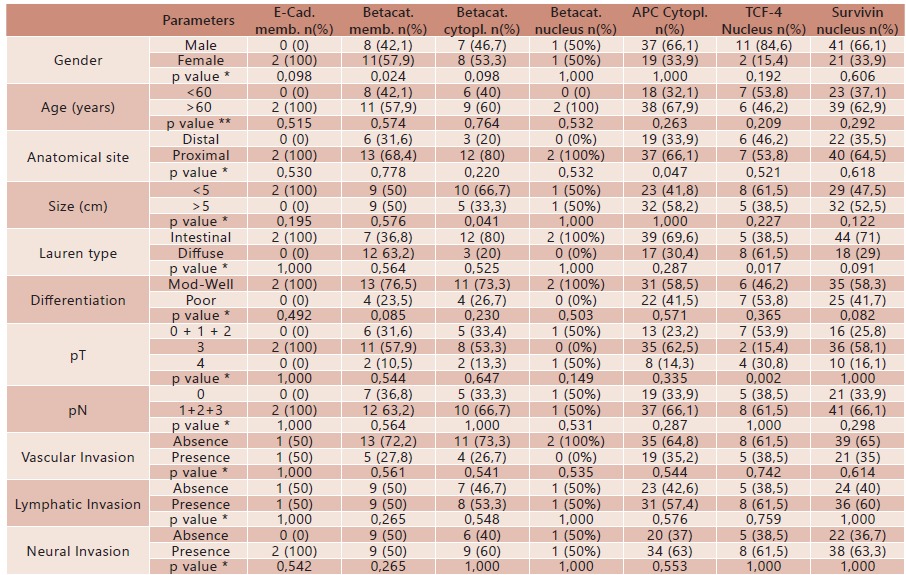
Relationship between clinico-pathological parameters and immunohistochemical expression ofE-cadherin, β-catenin, APC, TCF-4 and survivin in GC patients

E-cad.=E-cadherin; Betacat=betacatenin; APC=adenomatous polyposis coli; TCF-4=T-cell factor; Memb=membrane; Cytopl.=cytoplasm; n=number of cases; Mod-Well=moderate/well differentiate; Poor=poor differentiate; * Fisher test; ** Mann-Whitney test

## DISCUSSION

In the present study, was found a positive expression of E-cadherin at the membrane in only 3% of cases unrelated to the parameters analyzed. This result is lower than that described in the literature ranging from 32 to 60.9% and demonstrates the relationship with high-grade tumors and lymph node involvement^18, 20^. However, in the cytoplasm, was noted the expressed E-cadherin positive in 63.6% of cases. Thus, a hypothesis that may explain this difference found is that most of these studies conducted a joint analysis of E-cadherin expression in the membrane and cytoplasm.

Despite the low prevalence of E-cadherin in the membrane, was found the presence of betacatenin in the membrane in 29.7% of cases being more prevalent in females. Grabsch et al. reported the presence of betacatenin in 13.5% of cases and realized a combination of 93.2% between the simultaneous absence of betacatenin and E-cadherin in the membrane. However, Guerfali et al. found the prevalence of 61.3% and reported that the cases where there was loss of membrane expression of betacatenina and E-cadherin were associated with poorly differentiated tumors.

In the cytoplasm, was found the positive expression of betacatenin and APC in 23.4% and 94% of cases respectively. Betacatenin had relationship with tumors smaller than 5 cm and all proximal tumors showed positive expression of APC. The result obtained regarding APC was greater than that found for Ayed-Guerfali et al. (68.7%) that differently, showed no relationship with clinico-pathological data they studied. The finding of this sample suggests that the expression of APC protein was not involved in GC carcinogenesis, probably because their function was preserved without altering the functioning of the degradation complex, maintaining the betacatenin in the cytoplasm at physiological levels.

At the nucleus, was found the expression of betacatenin in 2 patients (3.1% of cases), with no significant relationship with the parameters analyzed, but the two were intestinal type by Lauren classification. Ayed-Guerfali et al. showed almost the same prevalence (3.75%), and using another evaluation criteria that considered abnormal the cases where betacatenin was not expressed or was expressed mainly in the nucleus and/or the cytoplasm, demonstrated the relationship between the abnormal pattern and patients in stages III and IV and with lymph node metastasis. Miyazawa et al. also found the expression of betacatenin only in intestinal type by Lauren and Grabsch et al. analyzing 401 gastric adenocarcinomas found that 6.5% of them had betacatenin strongly expressed in the nucleus and were associated with the intestinal type of Lauren. However, most authors reported a higher prevalence of betacatenin in GC, such as: Ohene-Abuakwa et al. 34%, Woo et al. 27%, and Yu et al. 15.1%. The variation found between the results probably occurs due to different testing methods and criteria used, as well as the discrepancy between the sample sizes of each study. Nevertheless, most of these studies could demonstrate a correlation between the betacatenin cellular expression and any clinico-pathological parameters, highlighting the importance of betacatenin in the GC tumorigenic process. However, the underlying molecular mechanisms leading to activation of the signaling pathway mediated by betacatenin still need to be clarified.

In relation to TCF-4 protein, this study showed positive expression in the nucleus in 19.4% of cases, values ​​below those reported by Yu et al. who were 86.5%. This difference probably is justified because we use more stringent criteria that only considered the expression positive when the score obtained by the product of the intensity and the area was greater than or equal to 4, while these authors considered positive when different from zero. Moreover, was observed relationship between the nuclear expression of TCF-4 and T4 tumors and diffuse type of Lauren tumors, while Yu et al. described relationship with the intestinal type. Is known that the nuclear TCF-4 has a major role in the last step of the canonical Wnt signaling by interacting with the nuclear betacatenin and trigger the transcription of target genes^25^. Thus, the low frequency of nuclear positive TCF-4 and betacatenin expression found in the present study suggests that the canonical pathway would not be involved in gastric carcinogenesis.

Survivin is a genetic target of the Wnt pathway and has its expression increased by betacatenin^4^. However, in this study was found no relationship between survivin and betacatenin expression, both in the nucleus and the cytoplasm. In this series survivin protein showed the same positive expression (93.9%) in the nucleus and the cytoplasm, and as Lu et al. has shown to be related to intestinal type tumors. Its molecular regulation mechanism is not well defined and there is controversy in the literature regarding its expression in GC. Song et al. analyzed 157 patients with stage III GC and found survivin nuclear expression in 40.1% of cases with predominance of tumors larger than 5 cm. Patients with negative immunoreactivity of survivin showed significantly higher survival^24^. Bury et al. found survivin expressed in 73.17% of patients and showed that carcinomas without survivin expression had lower incidence of distant metastasis and improved survival at 1, 2 and 3 years. Shintani et al. reported the presence of survivin in the cytoplasm andthe nucleus in 35% and 49% respectively. Moreover, they found a relationship between the nuclear expression of survivin and well/moderate differentiated gastric tumors. With the results found in this study, it can be reinforced the importance of survivin in GC carcinogenesis and suggest the possibility of it being stimulated by other pathway than the Wnt/betacatenin.

It´s known that the immunohistochemical evaluation offers several advantages but we are aware of the limitations of this approach. Changes in immunostaining are neither completely sensitive nor specific for changes in protein expression. Furthermore, immunohistochemistry does not provide direct information about the changed protein function. Studies examining the genetic and epigenetic events are needed.

However, in this study, the low frequency of positive E-cadherin expression in the membrane, without the consequent accumulation of betacatenin in the cytoplasm and/or nucleus, suggesting that the effect of E-cadherin protein does not induce activation of Wnt pathway. Furthermore, the high frequency of positive cytoplasmatic APC expression associated with low frequency of positive cytoplasmatic and nuclear betacatenin expression, suggesting a low prevalence of mutations in the APC gene in GC and betacatenin degradation via ubiquitination be occurring normally. Finally, the high frequency of nuclear survivin expression associated with low frequency of positive nuclear betacatenin and TCF-4 expressions, suggests that the canonical pathway was not activated in the GC, there is probably another parallel pathway responsible for the survivin production.

These results may contribute to a better understanding of this signaling pathway in gastric carcinogenesis and to identify possible targets such as the reduction of the survivin gene expression, for the development of drugs for GC treatment.

## CONCLUSION

Wnt/beta-catenin is not involved in gastric carcinogenesis. However, the high frequency of survivin allows to suggest that other signaling pathways must be involved in the transformation of gastric tissue.
